# COVID-19 Vaccine Rollout Strategies in Utah from Local Health Departments’ Perspectives: A Qualitative Analysis of Focus Group Discussions

**DOI:** 10.1089/heq.2024.0067

**Published:** 2025-01-13

**Authors:** Khanh N. C. Duong, Sajesh K. Veettil, Richard E. Nelson, Barbara E. Jones, Andrew T. Pavia, Danielle T. Nguyen, Makoto M. Jones, Matthew H. Samore, Susan L. Zickmund, Patrick Galyean, Nathorn Chaiyakunapruk

**Affiliations:** ^1^ Department of Pharmacotherapy, College of Pharmacy, University of Utah, Salt Lake City, Utah, USA.; ^2^ Department of Pharmacy Practice, School of Pharmacy, IMU University, Kuala Lumpur, Malaysia.; ^3^ IDEAS Center, Veterans Affairs Salt Lake City Health Care System, Salt Lake City, Utah, USA.; ^4^ Division of Epidemiology, Spencer Fox Eccles School of Medicine, University of Utah, Salt Lake City, Utah, USA.; ^5^ Division of Pulmonary & Critical Care, Spencer Fox Eccles School of Medicine, University of Utah, Salt Lake City, Utah, USA.; ^6^ Division of Pediatric Infectious Diseases, Spencer Fox Eccles School of Medicine, University of Utah, Salt Lake City, Utah, USA.

**Keywords:** COVID-19 vaccine strategies, mobile vaccine clinics, local health departments, Utah, focus group discussions, health equity

## Abstract

**Introduction:**

Local health departments (LHDs) play an essential role in providing COVID-19 vaccines to underserved populations in Utah. This study aimed to understand barriers to COVID-19 vaccine uptake for these populations and challenges faced by LHDs from LHDs’ perspectives. In addition, we explored LHDs’ experience with implementing COVID-19 mobile vaccine clinics (MVCs) in Utah.

**Materials and Methods:**

We conducted virtual focus group discussions (FGDs) from October 28 to November 1, 2022, with health officers from Utah’s Department of Health and Human Services (DHHS) and LHDs. We recruited participants via email, transcribed recordings verbatim, and analyzed data using inductive content analysis.

**Results:**

Eight participants, one from the Utah DHHS and seven from Utah’s LHDs (mostly executive directors or managers), participated in two FGDs. Barriers to vaccine uptake among underserved communities included structural, behavioral, and informational barriers. LHDs faced two main challenges to increasing vaccination rate: limited resources and the lack of established partnerships with trusted communities/organizations/leaders. Strategies implemented to increase vaccine uptake included multiple channels for vaccine access and information provision, and building multiple partnerships. Key lessons learned were the importance of partnerships with trusted community/organization leaders and building core staff for vaccine uptake. Regarding MVCs, they were effective in reaching underserved populations, however, their impact was unclear in rural areas.

**Conclusion:**

Building trust through partnerships with trusted community/organization leaders was crucial for increasing vaccine uptake in underserved populations and promoting health equity. The impact of MVCs on underserved populations in different settings remains unclear, further research is needed.

## Introduction

Health disparity in vaccine uptake is a critical issue in the COVID-19 vaccine rollouts in the United States, with minority populations receiving vaccines disproportionately compared with their population shares.^1–3^ Health equity, which ensures that everyone has a fair and just opportunity to access health care and achieve optimal health, is a central focus of public health policy.^4^ Enhancing equity in health care access requires improving across the entire spectrum of accessing health care, including the ability to identify health care needs, seek health care services, reach health care resources, use health care services, and be offered services appropriate to the needs for care, as defined in Levesque’s framework.^5^ In this article, we are considering equity in health care access in the “seeking health care services” dimension, including the physical existence of health services (providers) and the ability to reach health care services (patients).^5^ Using this perspective in accessing COVID-19 vaccine uptake in underserved populations, challenges and barriers arise from the demand side, communities’ barriers to obtaining vaccines, and the supply side, local health districts or health departments in implementing vaccine roll-outs in their communities.^5^

Several qualitative studies have explored communities’ barriers to vaccine uptake from their own perspectives.^6–8^ Exploring these barriers through local health departments’ (LHDs) perspectives is also equally important, as it ensures that communities are heard and strategies are implemented effectively to address their barriers. Communities’ perspective is the first piece of the puzzle. The remaining piece, LHDs’ challenges during vaccination implementation, is underexplored in the current literature. LHDs act as intermediaries between state health departments and local communities, therefore, understanding their challenges is crucial to planning practical and realistic strategies at the higher levels.

Utah is geographically characterized by a mix of urban centers and vast rural areas, where 77% of the state’s lands are in rural areas.^9^ There are 5 urban (100 or more persons per square mile [ppsm]), 12 rural [6 to 100 ppsm], and 12 frontier counties [6 or fewer ppsm] in Utah, which demonstrates significant variations in population density.^10^ In addition, there is an increasingly diverse population with the percentage of residents identified as a racial or ethnic minority increasing from 19.4% in 2010 to 24.6% in 2020.^11^ These demographic factors create unique challenges in reaching underserved populations with low vaccination rates.^12^

To increase vaccination in underserved populations, the state implemented multiple strategies, including mobile vaccine clinics (MVCs). This strategy aimed to bring vaccines to those in need, especially in rural areas with limited access to health care facilities. In the specific context of Utah, MVCs were run by LHDs or by the state health department through contracts with third parties. Understanding the experience of MVCs implementation through LHDs’ lens is valuable to evaluate the impact of this strategy and improve future efforts.

Therefore, this study aims to achieve two main objectives (1) to understand barriers and challenges to increasing COVID-19 vaccine uptake in underserved populations and (2) to explore the experience of implementing MVCs. Both objectives are through the LHDs’ perspectives.

## Materials and Methods

We carried out this qualitative study using focus group discussions (FGDs). Participants were LHDs officers who fulfilled one or more of the following criteria: (1) currently (or previously if during the COVID-19 pandemic) work at the Utah Department of Health and Human Services (DHHS) or LHDs, (2) were/are involved in the COVID-19 vaccine rollouts in Utah, and (3) were/are involved in the decision-making process for the COVID-19 vaccine rollouts in Utah. We planned to include representatives from the Utah DHHS because, first, they would provide insights into statewide strategies and the coordination efforts between state and LHDs; second, in Utah’s context, they were involved in implementing MVCs at the state level. Based on the study’s objectives, we selected participants purposively. We targeted recruited participants at the executive director’s level as they were directly responsible for vaccine rollouts and executed MVCs programs in their areas. We identified participants through referrals from senior policymakers in the state. We asked potential participants if they were interested in participating in online FGDs to share their experience with the COVID-19 vaccine rollout and MVCs at their LHDs. We contacted potential participants with up to three email attempts in seven working days. We sent the consent cover letter to participants with the recruitment emails so they could review it before agreeing to participate. Contact information was available in the consent cover letter and the recruitment information if potential participants had any questions. We obtained verbal consent at the beginning of the meetings.

With participants agreeing to participate, we contacted them to arrange times/dates for virtual meetings. We sent the FGDs guide and meeting agenda to all participants via email three to five days before the discussions. We conducted FGDs virtually through Zoom and each lasted 60 minutes. At least two researchers facilitated each discussion based on the FGDs guide (Supplementary Data S1), which comprised two parts with open-ended questions. In the first part, we asked participants about barriers and challenges to roll out COVID-19 vaccines in their areas. The discussion also explored the strategies used to reach underserved populations and any lessons learned. The second part focused on MVCs. Participants shared their experiences and perspectives on the effectiveness of these clinics and the challenges they faced. At the end of the discussions, we encouraged participants to provide additional information, if any.

We recorded each discussion and transcribed it immediately after the interview and perused it several times to understand the participants’ perspectives. We analyzed data using a qualitative content process to identify codes using inductive content analysis, which condensed ideas, phrases, or concepts into broader themes and subthemes.^13–15^ We approached external supervisors with experience in qualitative research to ensure the conformability of findings and coding agreement. The coding process was conducted using TAGUETTE software.^16^

The study was approved by The Ethics Board Committee at the University of Utah (The University of Utah Institutional Review Board) (IRB_00159803).

## Results

### General Characteristics of Participants

We conducted two FGDs with eight participants: one representative from Utah DHHS and seven from five of Utah’s 13 LHDs, two of whom are female. Participants’ roles varied, with four executive directors, one immunization bureau manager, one COVID event manager, and one public information officer. The LHDs involved TriCounty, Southeast Utah, Salt Lake County, Tooele County, and Central Utah, which cover 14 of 29 counties in Utah (Supplementary Data S2).

The themes identified were (1) Barriers to COVID-19 vaccine uptake for underserved populations, (2) Challenges faced by LHDs in increasing COVID-19 vaccine uptake in underserved populations, (3) COVID-19 vaccine rollout strategies, (4) Key lessons learned from implementing vaccine rollout strategies for underserved populations, and (5) Experience in implementing MVCs.

### Theme 1: Barriers to COVID-19 Vaccine Uptake for Underserved Populations

The barriers to COVID-19 vaccine uptake in Utah for underserved populations were grouped into three subthemes: Structural barriers, Behavioral barriers, and Informational barriers.

#### Subtheme 1.1: Structural barriers

Many participants identified access as a significant structural barrier to vaccine uptake. People living in rural areas without medical clinics or hospitals faced substantial difficulties getting vaccines. One participant said, *“We have some of our counties that have no medical clinics, hospitals, anything like that, and so we are pretty much the only ones that can provide some of those things there in those counties.”* In addition, transportation to get vaccines was a major challenge for them. One interviewee noted, “*Transportation was a major one.”*

#### Subtheme 1.2: Behavioral barriers

Mistrust in the government emerged as a key behavioral barrier to vaccine uptake. Participants noted that some people refused or hesitated to get vaccinated simply because the initiative was associated with the government. One director mentioned, “*When you bring the government into it, there’s already a stigma there that’s attached, says, we don’t need help, and we especially don’t need it from the government”*. Another added, *“No matter what efforts we’ve made there, some people just can’t convince them to take the vaccine. They’re already convinced that just because the government said to do it, that means they shouldn’t do it.*”

#### Subtheme 1.3: Informational barriers

Participants discussed several information barriers, including language barrier, lack of health education, and spreading of misinformation. One participant said that some people were turned away from vaccine sites because they did not understand English. One interviewee observed, “*Some of those underserved populations where English as a second language could have likely turned many people away.”* Misinformation and mistrust also posed considerable challenges in educating communities about the vaccine. One participant said, *“It becomes very difficult to do the education piece.”*

### Theme 2: Challenges Faced by LHDs in Increasing COVID-19 Vaccine Uptake in Underserved Populations

The challenges faced by LHDs in increasing COVID-19 vaccine uptake in underserved populations included two subthemes: Limited resources and Lack of established partnerships with trusted communities/organizations/leaders.

#### Subtheme 2.1: Limited resources

Participants highlighted challenges related to resource limitations, including the lack of freezers to store vaccines at the required temperature and staffing shortages. One participant shared, *“Not all the health departments had ultra-high cold freezers that would accommodate.”* Regarding human resources, many LHDs were short-staffed, which strained their ability to provide both routine services and vaccination efforts. One director shared, *“We were on basically the front line of response from the local health department side, our nursing director, me [executive director], our preparedness director, and a couple of others; we had basically no backup. So, we were working or on call all day, every day, for a long time.”* In addition, LHDs did not have enough bilingual staff or real-time translators. One said, *“We didn’t have, and still don’t have, a very large number of bilingual staff.”*

#### Subtheme 2.2: Lack of established partnerships with trusted communities/organizations/leaders

LHDs encountered challenges in reaching minority populations due to a lack of prior established relationships with community leaders. One shared, “*We were struggling to connect with our Hispanic community. We didn’t have any really great partner organizations. We reached out to a couple of Hispanic churches locally to see if they would like to sponsor vaccine events. Most of them did not want to.”*

### Theme 3: COVID-19 Vaccine Rollout Strategies

COVID-19 vaccine rollout strategies in Utah were categorized into three subthemes: Multiple channels for vaccine access, Multiple channels for vaccine information provision, and Multiple partnerships to facilitate vaccine access and information provision.

#### Subtheme 3.1: Multiple channels for vaccine access

LHDs implemented various vaccine rollout strategies to increase vaccine uptake by offering vaccines through multiple channels. These efforts targeted diverse populations by providing vaccines at mass vaccination sites, pharmacies, workplaces, schools, faith-based organizations, local markets, homeless shelters, senior centers, mobile vans, homes, and jails. One noted, *“Our senior population, aging is a part of the health department, so we were able to do outreach to our homebound and to the long-term care facilities that weren’t taken care of.”*

In areas lacking pharmacies, LHDs opened vaccination sites in popular markets frequently visited by underserved populations. One participant explained, *“We also worked with [Asian supermarkets] and some of the Latinx supermarkets, some of those supermarkets that don’t have pharmacies.”* To address transportation barriers, some counties provided free rides through Lyft or Uber to help people access vaccine clinics.

In addition, LHDs made vaccine registration easier and more accessible by offering walk-in and phone registration options, particularly effective for the elderly who were less likely to use online systems. One said, *“For the rollout to our elderly population, we had spent much time getting our online registration system up and going. And we realized that most of them wouldn’t use it. And so, we pivoted to walk in and phone calls.”*

Furthermore, to reduce fears among immigrant communities, some LHDs did not require identification (ID) cards or insurance for registering vaccines. One noted, *“We did get reports that some people were afraid we were asking for ID, so we were not asking for ID or insurance.”*

#### Subtheme 3.2: Multiple channels for vaccine information provision

LHDs emphasized that providing communities access to vaccine information was essential for increasing vaccine uptake. A public information officer described using multiple methods to provide vaccine information, including social media (such as Facebook), radio, newspapers, community newsletters, mail, and word of mouth. One director said that they tailored their communication strategies to the demographics of their counties. He shared, *“We did radio, we did newspaper, we did a lot of Facebook updates, Facebook was a great way to reach the northern half of the county. In the southern half of the county, down there, very few people had internet access in their homes. They could access the internet on their phone, but most were on prepaid plans or didn’t have a signal in their community. But it was a good way to reach them when they would come into town for groceries or those sorts of things.”* LHDs in rural counties valued the importance of traditional methods like mail, newsletters, and word of mouth over social media. He said, “*But mostly, we relied on word of mouth by reaching across all of those fronts. We would hope that the word would get out through word of mouth and social gatherings and churches, et cetera.”*

#### Subtheme 3.3: Multiple partnerships to facilitate vaccine access and information provision

All participants agreed that establishing multiple partnerships was a crucial strategy for facilitating vaccine access and providing vaccine information. Partnering with trusted community leaders and organizations was the key to increasing vaccine uptake in minority populations, as these entities are more trusted by their communities than LHDs. One participant said, *“It was a [health] vaccine clinic at their location, which was only a block away from our location. That was open to anybody in the community, so we ran under their banner. It was their vaccine clinic, but anyone in the community was welcome, and our nurses were there to help. Our staff was there to help direct traffic, and our nurses were there to help vaccinate.”* He further explained, “*Particularly when we would show up to do vaccine clinics, we don’t have an office there. So, we’re not super recognized. So, giving it to the [medical center], suddenly, our numbers jumped up. Because people wanted to go to their doctor or their physician assistant, that’s kind of an outline of how that worked for us.”* Another one said, *“We worked with our trusted community partners and leaders because they were the voices that a lot of these communities would listen to and understand and trust, so a lot of the times, we put these clinics in their hands to promote. We provided them with the resources that they needed.”*

An executive director highlighted a successful partnership with local businesses. He shared, *“I remember we had worked with the local Lowe’s Home Improvement store because they have a very large parking lot, and it’s very visible. We had pictures on our Facebook with our vaccine administrators and Lowe’s sign in the background. We even got a call from Lowe’s corporate saying that the best advertising they had that year was that story that went kind of viral about the first vaccines being opened to 12-year-olds. I think those partnerships with businesses were really successful. Most businesses were really supportive.”*

### Theme 4: Key Lessons Learned from Implementing Vaccine Rollout Strategies for Underserved Populations

Key lessons learned from implementing vaccine rollout strategies for underserved populations included two subthemes: Importance of partnerships with trusted community/organization leaders and Building core staff at LHDs for vaccine uptake.

#### Subtheme 4.1: Importance of partnerships with trusted community/organization leaders

All interviewees underscored the importance of partnerships with trusted community/organization leaders in increasing vaccine uptake among minority populations. These populations were closely connected with their leaders, making it more effective to reach them through these established relationships. One director said, “*They [minority populations] are very connected to the leaders of that population, and we basically do all of the legwork for getting clinics set up. We basically appeared as if they [community organizations] were going to give the vaccine, and we just happened to be the ones giving the shot in those situations. Because they were much more trusted than us [local health departments], that was one strategy that we identified was that we could just be in the background while some trusted organizations were the face of certain clinics*.”

#### Subtheme 4.2: Building core staff at LHDs for vaccine uptake

Several LHDs emphasized the essential role of building core staff to support vaccine uptake efforts. These staff members were trained to handle the logistical challenges of vaccine distribution, engage with diverse community representatives, and ensure equitable access to vaccines. One participant said, *“It’s important to have core staff to be able to run clinics. The lesson learned is that you can’t have a successful pandemic if you don’t have enough core staff to be able to run this overall.”* Another added, *“The group [core staff group] was established, and this group started having representation from all areas of Salt Lake County government.”*

### Theme 5: Experience in Implementing MVCs

Experience in implementing MVCs in Utah was categorized into three subthemes: Reaching underserved populations, Challenges in rural areas, and Doubts about effectiveness in rural areas.

#### Subtheme 5.1: Reaching underserved populations

Participants generally agreed that MVCs were effective in reaching underserved populations who might otherwise struggle to access vaccines. One participant shared, *“I think, just to share a perspective in our area, the pop-up vaccine-type clinics and mobile resources were really effective at certain times and with certain populations. For some of our very, very remote individuals, it was great to be able to bring the resources to them, and that helped.”* Another said, *“It’s very nice to talk about mobile pop-up clinics, and I do think that the community sees them as valuable. I think they increase convenience.”* Another added, *“But the occasional person that wouldn’t have been able to make it [get vaccine] to a regular site is incredibly thankful that you brought out that pop-up that makes it worth it. Even though they’re very small numbers.”*

#### Subtheme 5.2: Challenges in rural areas

MVCs in rural areas encountered significant challenges related to planning, turnout, and resource allocations. One participant highlighted the poor organization and communication surrounding these MVCs. He said, *“With X [a mobile van], X would come out and try to do vaccine clinics, but they were very bad at getting the word out. They would usually give us like 24 h’ notice if that. They’d just alert us and no one else. They’d alert us maybe eight hours in advance and say, “We’re gonna be in this parking lot tomorrow. “There was no rhyme or reason for it; no one ever knew. We would put the word out through the channels that we could. And then they would complain that they only had two people show up. Very poorly run from the state.”*

Another participant discussed the disappointment in turnout, which affected staff morale. He said, *“When we went out to some of these extreme frontier areas, we would expect to have a better response, and we may come out of there. Having done a clinic and giving out five to 10 doses at the most, we had to change our expectations, which constituted a successful clinic in one of those areas. We were driving three and a half hours one way out into the West desert to catch a very, very remote community. And we may only give a handful of doses. Then we get from one side of our district to the other, which is about a six-hour one-way trip. And that’s a pretty challenging thing to deal with when you’re looking at that big area and having people so spread out across there.”*

In addition, one interviewee mentioned the strain on staff resources. He said, *“When you’re planning these pop-up clinics and inside and out, and you’re dealing with the day-to-day stuff as well, who’s sick and who’s being quarantined and, the population of employees dealing with all these different elements.”*

#### Subtheme 5.3: Doubts about effectiveness in rural areas

Some LHDs directors, especially in rural and frontier counties, expressed doubts about the effectiveness of MVCs in their areas. While MVCs were seen as a tool to reach underserved populations and promote vaccine equity, their impact was perceived as limited and potentially inefficient. One said, *“Mobile vaccine clinics increased vaccine uptake, probably not as much as most people think.”* Another added, *“I don’t think we served very many in terms of the number of people at those sites that would not have served just as in our regular sites.”* Some participants also discussed the cost-effectiveness of MVCs. One said, *“But if you were just thinking about the cost/benefit ratio, it probably wouldn’t be worth it.”*

In addition, another participant argued that MVCs might be less effective in rural populations because residents in their areas are accustomed to traveling to town for essential services. He explained, *“I think the thing that you have to bear in mind, the very rural and frontier areas, is that everyone’s life depends on their ability to travel anyway. We don’t have populations of people that don’t have a means to travel because there are no services out here that you don’t have to drive to. You have to drive into town to get your groceries; you have to drive into town if you don’t have internet; you have to drive into town to pay your bills. So, if you don’t have access to a car or some network that allows you access to a car, you don’t live here. And so, I bring the vaccine to you versus having it close to the grocery store; it’s just as good to have it close to the grocery store as it is rather than to try to bring it out to you.”*

Some participants in rural areas suggested that MVCs might be more effective in urban areas. He said, *“I suspect mobile clinics in bigger areas will have been a bit more successful than ours.”*

A summary of themes and subthemes is presented in Table 1. Detailed quotes are available in Supplementary Data S3–S7. See Supplementary Data S8 for the definition of urban, rural, and frontier counties in Utah.

**Table 1.** Themes and Subthemes from Qualitative Data Analysis

**Theme 1**: Barriers to COVID-19 vaccine uptake for underserved populations • Subtheme 1.1: Structural barriers • Subtheme 1.2: Behavioral barriers • Subtheme 1.3: Informational barriers
**Theme 2:** Challenges faced by local health departments in increasing COVID-19 vaccine uptake in underserved populations • Subtheme 2.1: Limited resources • Subtheme 2.2: Lack of established partnerships with trusted communities/organizations/leaders
**Theme 3:** COVID-19 vaccine rollout strategies • Subtheme 3.1: Multiple channels for vaccine access • Subtheme 3.3: Multiple channels for vaccine information provision • Subtheme 3.3: Multiple partnerships to facilitate vaccine access and information provision
**Theme 4**: Key lessons learned from implementing vaccine rollout strategies for underserved populations • Subtheme 4.1: Importance of partnerships with trusted community/organization leaders • Subtheme 4.2: Building core staff at local health departments for vaccine uptake
**Theme 5:** Experience in implementing mobile vaccine clinics • Subtheme 5.1: Reaching underserved populations • Subtheme 5.2: Challenges in rural areas • Subtheme 5.3: Doubts about effectiveness in rural areas

## Discussion

The barriers to COVID-19 vaccine access for underserved populations from LHDs’ views discussed in this study included structural barriers (limited clinic availability and transportation issues), behavioral barriers (mistrust in government or health systems), and informational barriers (language barriers, misinformation about the vaccine, and low health literacy). These factors significantly complicate efforts to increase vaccine uptake in these communities. Similar barriers have been identified in qualitative studies involving residents of these communities,^6–8,17^ indicating that LHDs were well aware of these communities’ obstacles. As a result, LHDs implemented multi-pronged strategies to address these barriers, as highlighted in Theme 3 in the results. An interesting point, which did not emerge during the first part of the discussion (i.e., challenges and barriers during vaccine uptake) but in the second part on MVCs, is that some participants from rural and frontier counties perceived that living in rural areas was a personal choice rather than a disadvantage, particularly regarding transportation. They argued that because they chose to live in rural areas, they owned cars for commuting. This perspective might suggest a potential disconnect between the communities’ experiences and the LHDs’ understanding of their communities’ challenges.

LHDs faced two primary challenges in their vaccination programs: resources and trust. The former may be easier to address than the latter. Resource limitations included material shortages (e.g., freezers for vaccines) and labor shortages (e.g., staff for vaccine outreach, bilingual staff). These issues, which have been highlighted as significant problems for health centers in COVID-19 vaccination by the Kaiser Family Foundation,^18^ are particularly severe in rural health districts. Resource shortages especially affect underserved communities more severely as they rely heavily on local health districts. One short-term solution, as done by the state health department in Utah, was to provide state support through third-party MVCs, which helped alleviate staff and equipment shortages during the COVID-19 vaccination phase. However, long-term solutions require more innovative and sustainable approaches.

The lack of established relationships with trusted community organizations is a more challenging issue. Mistrust in the health system makes it difficult to reach underserved communities, provide accurate vaccine information, and convince them to get vaccinated. Mistrust is a central issue in vaccine hesitancy, as highlighted in several studies.^6–8^ This mistrust may stem from various complex factors, including systemic racism, hostility toward immigrants, a belief that drug companies and the government prioritize profit over community health, and personal negative experiences with the health care system.^6–8^ Building trust is a difficult but highly rewarding task. Trust in communities could be built through trusted community or organization leaders. These trusted entities serve as effective bridges between health departments and their local communities. Numerous studies have demonstrated the critical role of trusted partners in promoting vaccine uptake.^6,19–21^ Building and maintaining these partnerships are essential for narrowing health care inequity in these communities.^22^ We must earn and save this trust savings account so we can use it in future crises. This is a key lesson learned.

Regarding the experience of MVCs implementation, LHDs generally agreed that this strategy effectively reached underserved populations. Even though turnout might be lower than expected, they continued to implement MVCs, considering the equity aspect. LHDs in rural and frontier areas, however, expressed doubts about the effectiveness of MVCs compared to urban areas. Their concern may stem from their definition of MVCs from a cost-effectiveness perspective. The discussion revealed that they measured the effectiveness of MVCs by the number of vaccines administered relative to the efforts and costs involved. This is a common definition and metric for the cost-effectiveness of an intervention.^23^ However, equity-focused interventions, like MVCs, require a broader definition and measure of effectiveness that includes effectiveness, cost, and equity in the same equation. Equity should not be merely one of the multiple criteria in decision-making; rather, it should be explicitly integrated into the cost-effectiveness evaluation, called equity-informative economic evaluations by some authors,^24–26^ providing more measurable and stronger evidence for decision-makers.^27^ Besides that, it is also reasonable to question the effectiveness of MVCs in different settings, as literature primarily examined their impact in urban areas.^28–30^ Further studies are needed to address this gap.

This study has several limitations. First, health equity, in our work, focused on equitable opportunities for health care access, so it solely explored the barriers and challenges related to accessing care. We acknowledge that there are other definitions and frameworks of health equity that dive deeper into the root causes of health inequity,^31,32^ particularly in the context of the COVID-19 vaccination. However, given the practical orientation of our approach and our targeted audience of public health practitioners and policymakers, this focus on health equity is more suitable. Second, we could include only eight participants, which might not capture the full range of experiences and perspectives of all LHDs in Utah. However, most of them are executive directors or managers, which gave us valuable and profound insights from people who directly executed and ran the COVID-19 vaccine program. Third, we did not quantify the frequency of barriers and challenges cited. Four, conducting the study within Utah might limit its generalizability. However, given these participants’ vital role in vaccine implementation, the in-depth practical experiences provided could help inform future research and policy efforts in other states in the United States.

## Conclusion

This study provided information on barriers and challenges to vaccine uptake in underserved populations and lessons learned during this process from perspectives of LHDs. Building and maintaining partnerships with trusted communities, organizations, and leaders was essential in increasing vaccine uptake in these populations. The practical experiences with COVID-19 MVCs also varied between urban and rural areas, which should be investigated further. Overall, this information can support policymakers in developing more effective and equitable vaccine rollout plans by considering the experiences and insights of those who have already implemented them.

## Data Availability

The data that support the findings of this study are provided upon reasonable request of researchers to the corresponding author (N.C.; nathorn.chaiyakunapruk@utah.edu).

## Abbreviations Used

DHHS: Department of Health and Human Services
FGDs: Focus Group Discussions
LHDs: Local Health Departments
MVCs: Mobile Vaccine Clinics
