# Supplementary material for: COVID-19 Vaccine Rollout Strategies in Utah from Local Health Departments’ Perspectives: A Qualitative Analysis of Focus Group Discussions
Source: Health Equity. 2025 Jan 13;9(1):31–40. doi: 10.1089/heq.2024.0067 (PMC12290390; doi:10.1089/heq.2024.0067)
Supplement: Supplementary Data S1 [file heq.2024.0067_supp_datas1.docx]

**SUPPLEMENTARY MATERIAL**

**COVID-19 vaccine rollout strategies in Utah from local health departments’ perspectives: A qualitative analysis of focus group discussions**

# Supplementary S1: Focus group discussions/interviews moderator guide

| **Focus Group Discussion Questions Guide** |
| --- |
| **Introduction:**   - Could you please introduce yourself and your positions during COVID-19 vaccine rollout programs?   **Part 1: COVID-19 Vaccine Rollout Strategies across Utah**   - Can you please share with us which strategies your Local Health Department (LHD) was/ are conducting to roll out COVID-19 vaccines? - Can you please describe the characteristics of people in your local community and county? For example, race/ethnicity, socioeconomic status, or rural/urban areas? - How did your LHD distribute and educate about the COVID-19 vaccination to underserved populations? Are there specific strategies you launched for these populations? - What challenges did you face in implementing these strategies? - What, if anything, would you have done differently if you could?   **Part 2: COVID-19 mobile vaccine clinics in Utah**   - Did your LHD implement mobile/pop-up vaccine clinics? What types of mobile/pop-up clinics are in your setting? - What do you think about the good things, bad things, or challenges of the pop-up vaccine clinics in your setting? - Are mobile vaccine clinics increasing vaccine uptake in your targeted populations? How does that compare to other strategies, such as stationary clinics (pharmacies, physician clinics, hospitals, etc.)? |
