# Supplementary material for: COVID-19 Vaccine Rollout Strategies in Utah from Local Health Departments’ Perspectives: A Qualitative Analysis of Focus Group Discussions
Source: Health Equity. 2025 Jan 13;9(1):31–40. doi: 10.1089/heq.2024.0067 (PMC12290390; doi:10.1089/heq.2024.0067)
Supplement: Supplementary Data S2 [file heq.2024.0067_supp_datas2.docx]

**SUPPLEMENTARY MATERIAL**

**COVID-19 vaccine rollout strategies in Utah from local health departments’ perspectives: A qualitative analysis of focus group discussions**

# Supplementary S2: Local Health Departments participated in the study

| **Local Health Department** | **Counties covered by Local Health Department** | **Number of participants** | **Participants’ Positions** |
| --- | --- | --- | --- |
| Utah Department of Health | State Level | 1 | COVID-19 Immunization Program Manager |
| Salt Lake County Health Department | Salt Lake | 3 | - Immunization Bureau Manager - COVID event manager - Public Information Officer |
| Tooele County Health Department | Tooele | 1 | Executive Director/ Health Officer |
| Central Utah Public Health Department | Juab, Millard, Piute, Sanpete, Sevier, and Wayne | 1 | Executive Director/ Health Officer |
| TriCounty Health Department | Daggett, Duchesne, and Uintah | 1 | Executive Director/ Health Officer |
| Southeast Utah Health Department | Carbon, Emery, and Grand | 1 | Executive Director/ Health Officer |


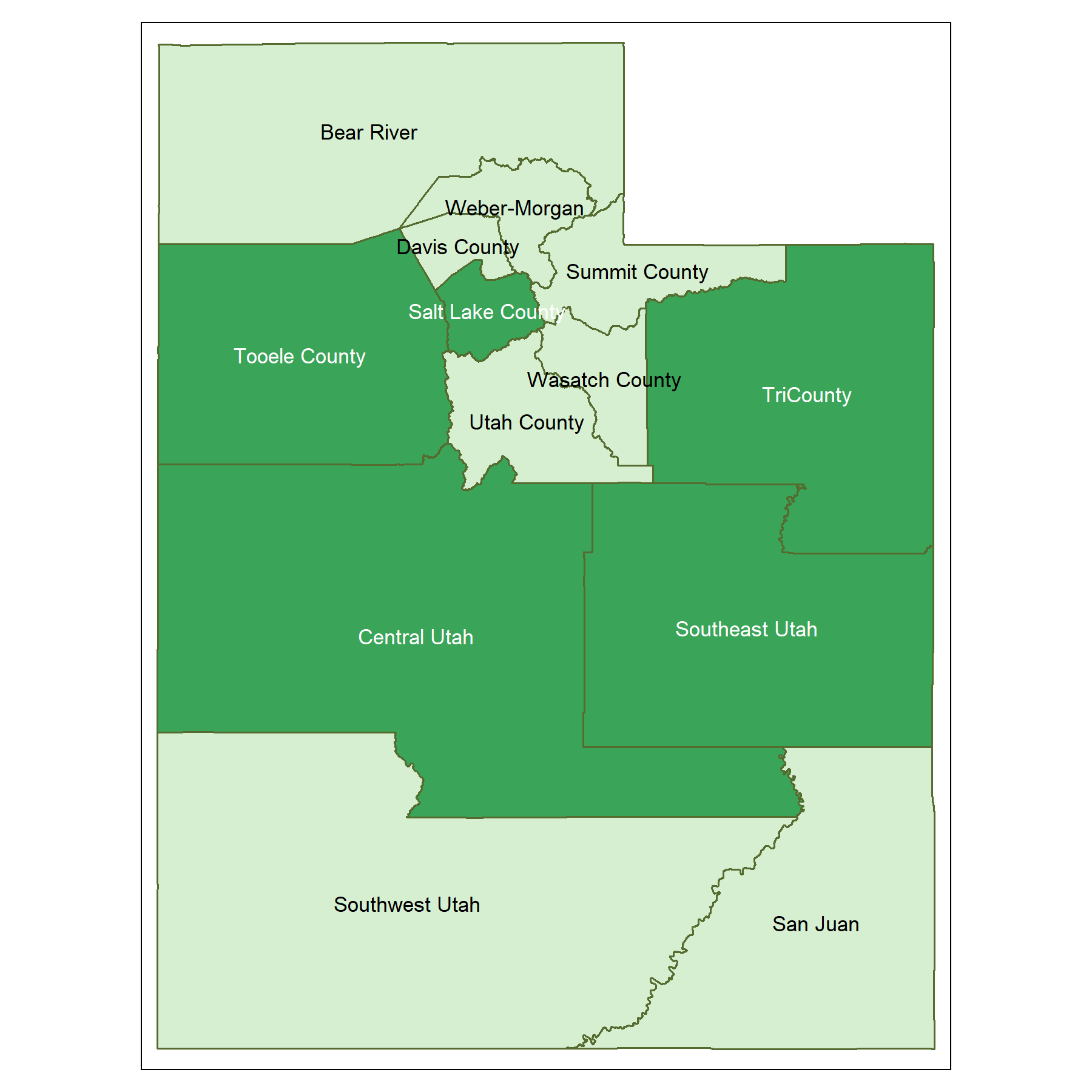


Thirteen Local Health Departments (LHDs) in Utah (LHDs participating in the focus group discussion are presented in dark green).
