# Supplementary material for: COVID-19 Vaccine Rollout Strategies in Utah from Local Health Departments’ Perspectives: A Qualitative Analysis of Focus Group Discussions
Source: Health Equity. 2025 Jan 13;9(1):31–40. doi: 10.1089/heq.2024.0067 (PMC12290390; doi:10.1089/heq.2024.0067)
Supplement: Supplementary Data S3 [file heq.2024.0067_supp_datas3.docx]

**SUPPLEMENTARY MATERIAL**

**COVID-19 vaccine rollout strategies in Utah from local health departments’ perspectives: A qualitative analysis of focus group discussions**

# Supplementary S3: Theme 1: Barriers to COVID-19 vaccine uptake for underserved populations

| **Sub-theme** | | **Quotes** |
| --- | --- | --- |
| Sub-theme 1.1: Structural barriers | | |
|  | Inconvenience in accessing health services in terms of travel and hours of services | - “Another thing that happened during the response that was impactful is there were complications in the rural areas that did not have the U of U or IHC clinics.” - “We have some of our counties that have no medical clinics, hospitals, anything like that, and so we are pretty much the only ones that can provide some of those things there in those counties.” |
|  |  | - “Transportation was a major one.” |
| Sub-theme 1.2: Behavioral barriers | | |
|  | Mistrust | - “A big one was mistrust in government.” - “Because of distrust of government, public health” - “When you bring the government into it, there's already a stigma there that's attached, says, we don't need help, and we especially don't need it from the government.” - “No matter what efforts we've made there, some people just can't convince them to take the vaccine. They're already convinced that it's a bad thing. They're already convinced that just because the government said to do it, that means they shouldn't do it.” |
| Sub-theme 1.3: Informational barriers | | |
|  | Language barrier | - “Language was definitely one.” - “Some of those underserved populations where English as a second language could have likely turned many people away.” |
|  | Health education | - “It becomes very difficult to do the education piece.” - “The education piece, I think, is what's most important.” |
|  | Misinformation | - “Misinformation, that becomes very difficult" - “Misinformation, especially later in the vaccination, when things had slowed down a little bit, and everyone who wanted the vaccine seemingly had already gotten it. A big part of what we had to work on was combating this information.” |
