# Supplementary material for: COVID-19 Vaccine Rollout Strategies in Utah from Local Health Departments’ Perspectives: A Qualitative Analysis of Focus Group Discussions
Source: Health Equity. 2025 Jan 13;9(1):31–40. doi: 10.1089/heq.2024.0067 (PMC12290390; doi:10.1089/heq.2024.0067)
Supplement: Supplementary Data S4 [file heq.2024.0067_supp_datas4.docx]

**SUPPLEMENTARY MATERIAL**

**COVID-19 vaccine rollout strategies in Utah from local health departments’ perspectives: A qualitative analysis of focus group discussions**

# Supplementary S4: Theme 2: Challenges faced by local health departments in increasing COVID-19 vaccine uptake in underserved populations

| **Sub-theme** | | **Quotes** |
| --- | --- | --- |
| Sub-theme 2.1: Limited resources | | |
|  | Limited freezers to store vaccines | - “Not all the health departments had ultra-high cold freezers that would accommodate.” |
|  | Insufficient staff | - “We pretty much shut down most of our public health services to staff everything we did from case investigation to contact tracing to vaccination.” - “We didn't have and still don't have, a very large number of bilingual staff. It would be very difficult for us to schedule our clinics and maybe do an outreach clinic simultaneously.” - “We always struggled with real-time translators at all sites. We did not have staff that could manage that.” - “A very small minority of our population might speak an obscure language, and we run into issues when someone would come to a clinic and wish that we had had better translation services at the moment. But sometimes that wasn't possible.” - “We were on basically the front line of response from the local health department side, our nursing director, me [executive director], our preparedness director, and a couple of others; we had basically no backup. So, we were working or on call all day, every day, for a long time.” |
| Sub-theme 2.2: Lack of established partnerships with trusted communities/organizations/leaders | | |
|  | Difficulty in reaching out to target communities due to lack of prior relationship with community leaders | - “We were struggling to connect with our Hispanic community. We didn't have any really great partner organizations. We reached out to a couple of Hispanic churches locally to see if they would like to sponsor vaccine events. Most of them did not want to.” |
