# Supplementary material for: COVID-19 Vaccine Rollout Strategies in Utah from Local Health Departments’ Perspectives: A Qualitative Analysis of Focus Group Discussions
Source: Health Equity. 2025 Jan 13;9(1):31–40. doi: 10.1089/heq.2024.0067 (PMC12290390; doi:10.1089/heq.2024.0067)
Supplement: Supplementary Data S5 [file heq.2024.0067_supp_datas5.docx]

**SUPPLEMENTARY MATERIAL**

**COVID-19 vaccine rollout strategies in Utah from local health departments’ perspectives: A qualitative analysis of focus group discussions**

# Supplementary S5: Theme 3: COVID-19 vaccine rollout strategies

| **Sub-theme** | | **Quotes** |
| --- | --- | --- |
| Sub-theme 3.1: Multiple channels for vaccine access | | |
|  | Multiple types of vaccine clinics: mass vaccine clinics, vaccines at pharmacies, vaccines at workplaces, vaccines at schools, vaccines at faith-based organizations, vaccines at local markets, vaccines at homeless shelters, vaccines at senior centers, vaccine mobile vans, vaccines at home, vaccines at jails | - “We set up large indoor mass vaccine clinics.” - “We also worked with [Asian supermarkets] and some of the Latinx supermarkets, some of those supermarkets that don't have pharmacies.” - “Our senior population, aging is a part of the health department, so we were able to do outreach to our homebound and to the long-term care facilities that weren't taken care of.” - “We had a mobile clinic that we would take out to some of the more outlying communities.” - “We did churches, we did businesses, we did food pantry events, Hispanic supermarkets without pharmacies where there was no COVID vaccine available, those Hispanic markets didn't have pharmacies.” - “We also do vaccination at the jail and the homeless shelters. We have mental health facilities; for those who normally won't come and get vaccines, we will go to them.” |
|  | Multiple types of supported transportation to vaccine sites | - “We provided free Lyft or Uber rides to the locations.” |
|  | Multiple types of ease of vaccine registration (online/ walk-in/ phone-call appointment; no require ID or insurance) | - “We initially had online appointments, then we made walk-in appointments for people that couldn't get online.” - “For the rollout to our elderly population, we had spent much time getting our online registration system up and going. And we realized that most of them wouldn't use it. And so, we pivoted to walk in and phone calls.” - “We didn't ask for Identification cards (IDs) at these clinics.” - “We did get reports that some people were afraid we were asking for ID, so we were not asking for ID or insurance.” |
| Sub-theme 3.2: Multiple channels for vaccine information provision | | |
|  | Provide vaccine information through social media, radio, newspapers, community newsletters, mail, or by mouth. | - “We did radio, we did newspaper, we did a lot of Facebook updates; Facebook was a great way to reach the northern half of the county. In the southern half of the county, down there, very few people had internet access in their homes. They could access the internet on their phone, but most were on prepaid plans or didn't have a signal in their community. But it was a good way to reach them when they would come into town for groceries or those sorts of things.” - “We did many community newsletters. But mostly, we relied on word of mouth by reaching across all of those fronts. We would hope that the word would get out through word of mouth and social gatherings and churches, et cetera.” - “Their community newsletter was the best way to reach people in that area.” - “We did much more education through direct mailers that went out to every PO Box.” - “I think the direct mailer with clear, concise information was pretty valuable later. That would've been very difficult to do early on because things were changing so rapidly. Sending out direct mailers with accurate information we felt was helpful.” |
| Sub-theme 3.3: Multiple partnerships to facilitate vaccine access and information provision | | |
|  | Partner with trusted communities/ organizations to reach out to underserved populations | - “Partnering with the Multi-Multicultural Center” - “It was a [health] vaccine clinic at their location, which was only a block away from our location. That was open to anybody in the community, so we ran under their banner. It was their vaccine clinic, but anyone in the community was welcome, and our nurses were there to help. Our staff were there to help direct traffic, and our nurses were there to help vaccinate.” - “Particularly, when we would show up to do vaccine clinics, we don't have an office there. So, we're not super recognized. So, giving it to the [medical center], suddenly, our numbers jumped up. Because people wanted to go to their doctor or their physician assistant. That's kind of an outline of how that worked for us.” - “There was a clinic there, and we partnered with them. They were able to really promote the vaccine and offer vaccines in some of the more rural areas. And in fact, they had community health workers taking vaccines out to shut-in people, et cetera. We let them do whatever they thought was best on the reservation.” - “We worked with our trusted community partners and leaders because they were the voices that a lot of these communities would listen to and understand and trust, so a lot of the times, we put these clinics in their hands to promote. We provided them with the resources that they needed. We would create flyers for them in multiple different languages.” - “We also worked with some of our tribal organizations.” |
|  | Partner with businesses to have convenient locations to operate vaccine clinics | - “We worked with some area businesses to secure locations we felt would be better.” - “So, when we'd have a rollout to 12-year-olds, we picked a location, we worked with a business usually, on a location that we felt like was a good location for the drive-through clinic that wouldn't back up onto a major roadway or things like that. Ensure the logistics made sense, and then we would repeat the process”. - “I remember we had worked with the local Lowe's Home Improvement store because they have a very large parking lot, and it's very visible. We had planned a mass vaccine event there. We had pictures on our Facebook with our vaccine administrators and Lowe's sign in the background. We even got a call from Lowe's corporate saying that the best advertising they had that year was that story that went kinda viral about the first vaccines being opened to 12-year-olds. Lots of people picked up on that. So, generally, I think those partnerships with businesses were really successful. Most businesses were really supportive.” |
