# Supplementary material for: COVID-19 Vaccine Rollout Strategies in Utah from Local Health Departments’ Perspectives: A Qualitative Analysis of Focus Group Discussions
Source: Health Equity. 2025 Jan 13;9(1):31–40. doi: 10.1089/heq.2024.0067 (PMC12290390; doi:10.1089/heq.2024.0067)
Supplement: Supplementary Data S6 [file heq.2024.0067_supp_datas6.docx]

**SUPPLEMENTARY MATERIAL**

**COVID-19 vaccine rollout strategies in Utah from local health departments’ perspectives: A qualitative analysis of focus group discussions**

# Supplementary S6: Theme 4. Key lessons learned from implementing vaccine rollout strategies for underserved populations

| **Sub-theme** | **Quotes** |
| --- | --- |
| Sub-theme 4.1: Importance of partnerships with trusted community/ organization leaders | |
|  | - “They [minority populations] are very connected to the leaders of that population, and we basically do all of the legwork for getting clinics set up. We basically appeared as if they [community organizations] were going to give the vaccine, and we just happened to be the ones giving the shot in those situations. Because they were much more trusted than us [local health departments]. That was one strategy that we identified was that we could just be in the background while some trusted organizations were the face of certain clinics. We ran under their banner. It was their vaccine clinic, but anyone in the community was welcome, and our nurses were there to help. Our staff were there to help direct traffic, and our nurses were there to help vaccinate.” - “One of the big successes was that we have federally qualified health care facilities. And, we partnered with them, one's in [place], and one is in [place], and they touch a lot in [place] which is an underserved community. We can transfer vaccines to them because they have a population that trusts them. But we felt like moving some of our allotment to them would help to reach a population that we normally wouldn't reach.” - “And, early on, we identified key community partners and individuals that were tied into the community.” - “Working with employers is important to do. Identifying those players that have a lot of ethnic or racial groups or other high-risk groups, low-income, or vulnerable populations. Being able to deliver those have clinics at their places of employment were very critical.” |
| Sub-theme 4.2: Building core staff at local health departments for vaccine uptake | |
|  | - “The group [core staff group] was established, I think, in January or February of 2021; this group started having representation from all areas of Salt Lake County government. We had folks represented by the Office of New Americans, the Office of Diversity and Equity, folks from the mayor's office, folks from the health department. This group intended to look at ways that we can make the mass vaccination sites more welcoming, so that looked like doing walkthroughs, making/creating recommendations on how we can implement better training for cultural awareness, and other aspects to just make those sites more welcoming, to folks that were attending.” - “Later on, this group [core staff group] was responsible for distributing grant funding to about 15 to 20 local community-based organizations working to serve various communities across Salt Lake County, the Black community, the Latinx community, and the Pacific Islander community. - “We have a core staff that knows what they're doing regarding vaccines. When you have core staff, we have our core nurses who can make sure that the vaccines are in the right temperatures.” - “It's important to have core staff to be able to run clinics. I mean, not just mass clinic, but outreach, too, which was really important. The lesson learned is that you can't have a successful pandemic if you don't have enough core staff to be able to run this overall.” |
