# Supplementary material for: COVID-19 Vaccine Rollout Strategies in Utah from Local Health Departments’ Perspectives: A Qualitative Analysis of Focus Group Discussions
Source: Health Equity. 2025 Jan 13;9(1):31–40. doi: 10.1089/heq.2024.0067 (PMC12290390; doi:10.1089/heq.2024.0067)
Supplement: Supplementary Data S7 [file heq.2024.0067_supp_datas7.docx]

**SUPPLEMENTARY MATERIAL**

**COVID-19 vaccine rollout strategies in Utah from local health departments’ perspectives: A qualitative analysis of focus group discussions**

# Supplementary S7: Theme 5: Experience in implementing mobile vaccine clinics

| **Sub-theme** | **Quotes** |
| --- | --- |
| Sub-theme 5.1: Reaching underserved populations | |
|  | - “I think, just to share a perspective in our area, the pop-up vaccine-type clinics and mobile resources were really effective at certain times and with certain populations. For some of our very, very remote individuals, it was great to be able to bring the resources to them, and that helped.” - “It's very nice to talk about mobile pop-up clinics, and I do think that the community sees them as valuable. I think they increase convenience.” - “But the occasional person that wouldn't have been able to make it [get vaccine] to a regular site is incredibly thankful that you brought out that pop-up that makes it worth it. Even though they're very small numbers.” - “Every mobile clinic you do, there's somebody or a couple of people that you wouldn't have gotten some way. And so that's valuable, particularly when people are so worried.” - “I feel like it was still worth it to do mobile vaccine clinics.” |
| Sub-theme 5.2: Challenges in rural areas | |
|  | - “With X [a mobile van], X would come out and try to do vaccine clinics, but they were very bad at getting the word out. They would usually give us like 24 hours' notice if that. They'd just alert us and no one else. They'd alert us maybe eight hours in advance and say, "We're gonna be in this parking lot tomorrow." There was no rhyme or reason for it; no one ever knew. We would put the word out through the channels that we could. And then they would complain that they only had two people show up. Very poorly run from the state.” - “I don't mean to pile on X [a mobile van], but we had a couple of instances stand out where they had reached out to our school districts to do vaccine clinics at our schools once students became eligible. They were done by preregistration, so we knew how many kids would be there, but they didn't even bring enough vaccines to cover what the people had registered. So, we [the local health department] had to fill in for them. That's just a great fond memory.” - “Every interaction I had with X [a mobile van] was just really poor. It was always very poor planning, a very poorly thought-out process with very low value added to our efforts.” - “You'd set up a pop-up clinic, even if it were well advertised, and two people would show up. And you'd feel like you wasted your time. And sometimes they'd show up, and you'd be really busy. Challenges are just maintaining staff enthusiasm, particularly as it continued to drag on and on.” - “Our nurses themselves, I think we managed their load. They could rotate in and out, so they didn't get burned out quite as quickly. When you're planning these pop-up clinics and inside and out, and you're dealing with the day-to-day stuff as well, who's sick and who's being quarantined and, the population of employees dealing with all these different elements. That's a challenge. I mean, it's not again a clinic itself, but managing everything else that was going on with the disease while still rolling out the vaccine.” - “We had to change our expectations early on. When we went out to some of these extreme frontier areas, we would expect to have a better response, and we may come out of there. Having done a clinic and giving out five to 10 doses at the most, we had to change our expectations, which constituted a successful clinic in one of those areas. We were driving three, three, and a half hours one way out into the West desert to catch a very, very remote community. And we may only give a handful of doses. And then we get from one side of our district to the other, about a six-hour one-way trip. And that's a pretty challenging thing to deal with when you're looking at that big area and having people so spread out across there.” - “To be honest with you, we can't sustain the way we are going in just having government, trying to run it because there's much burnout. Another thing, it's like we [local health departments] don't, the government don't always have the resources.” |
| Sub-theme 5.3: Doubts about effectiveness in rural areas | |
|  | - “Mobile vaccine clinics increased vaccine uptake, probably not as much as most people think.” - “I don't think we served very many in terms of the number of people at those sites that would not have served just as in our regular sites.” - “But if you were just thinking about cost/benefit ratio, it probably wouldn't be worth it.” - “There was a Hispanic LDS ward who wanted to sponsor a vaccine. They wanted to do it in the evening. They assured us that they had 40 or 50 families that they had communicated with that they felt would show up. We went, did that, and did two vaccines.” - “I suspect mobile clinics in bigger areas will have been a bit more successful than ours.” - “I think the thing that you have to bear in mind, the very rural and frontier areas, is that everyone's life depends on their ability to travel anyway. We don't have populations of people that don't have a means to travel because there are no services out here that you don't have to drive to. You have to drive into town to get your groceries; you have to drive into town if you don't have internet; you have to drive into town to pay your bills. So, if you don't have access to a car or some network that allows you access to a car, you don't live here. And so, I bring the vaccine to you versus having it close to the grocery store; it's just as good to have it close to the grocery store as it is rather than to try to bring it out to you.” |
