# Supplementary material for: COVID-19 Vaccine Rollout Strategies in Utah from Local Health Departments’ Perspectives: A Qualitative Analysis of Focus Group Discussions
Source: Health Equity. 2025 Jan 13;9(1):31–40. doi: 10.1089/heq.2024.0067 (PMC12290390; doi:10.1089/heq.2024.0067)
Supplement: Supplementary Data S8 [file heq.2024.0067_supp_datas8.docx]

**SUPPLEMENTARY MATERIAL**

**COVID-19 vaccine rollout strategies in Utah from local health departments’ perspectives: A qualitative analysis of focus group discussions**

**Supplementary S8**: County Classifications in Utah

| **County Classification** | **Counties** |
| --- | --- |
| **Frontier**  (6 or fewer persons per square mile) | Beaver, Daggett, Emery, Garfield, Grand, Juab, Kane, Millard, Piute, Rich, San Juan, Wayne |
| **Rural**  (6 to 100 persons per square mile) | Box Elder, Carbon, Duchesne, Iron, Morgan, Sanpete,Sevier, Summit, Tooele, Uintah, Washington, Wasatch |
| **Urban**  (100 or more persons per square mile) | Cache, Davis, Salt Lake, Utah, Weber |

Reference: U.S. Department of Health and Human Services, 2021, Overview of the State - Utah – 2021, link: <https://mchb.tvisdata.hrsa.gov/Narratives/Overview/b659aee2-3530-4e9f-ba55-07f91d6cf75f> [Accessed 2024/08/26]
